# Supplementary material for: Delayed generation of functional virus-specific circulating T follicular helper cells correlates with severe COVID-19
Source: Nat Commun. 2023 Apr 15;14:2164. doi: 10.1038/s41467-023-37835-9 (PMC10105364; doi:10.1038/s41467-023-37835-9)
Supplement: Supplementary file 1 — Supplementary Information [file 41467_2023_37835_MOESM1_ESM.pdf]

Supplementary figure 1

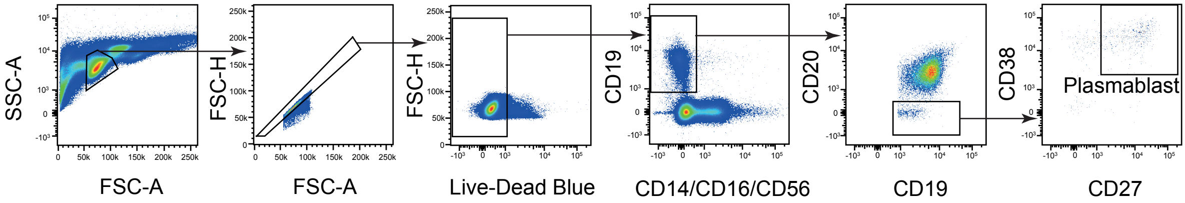

Supplementary Figure 1 Representative plasmablast gating strategy. Representative gating strategy to identify plasmablast in PBMC by flow cytometry. B cells were identified with lineage (CD14/CD16/CD56) negative but CD19 positive population. Within CD20 negative population, cells expressing CD27 and CD38 were identified as plasmablast.

Supplementary figure 2

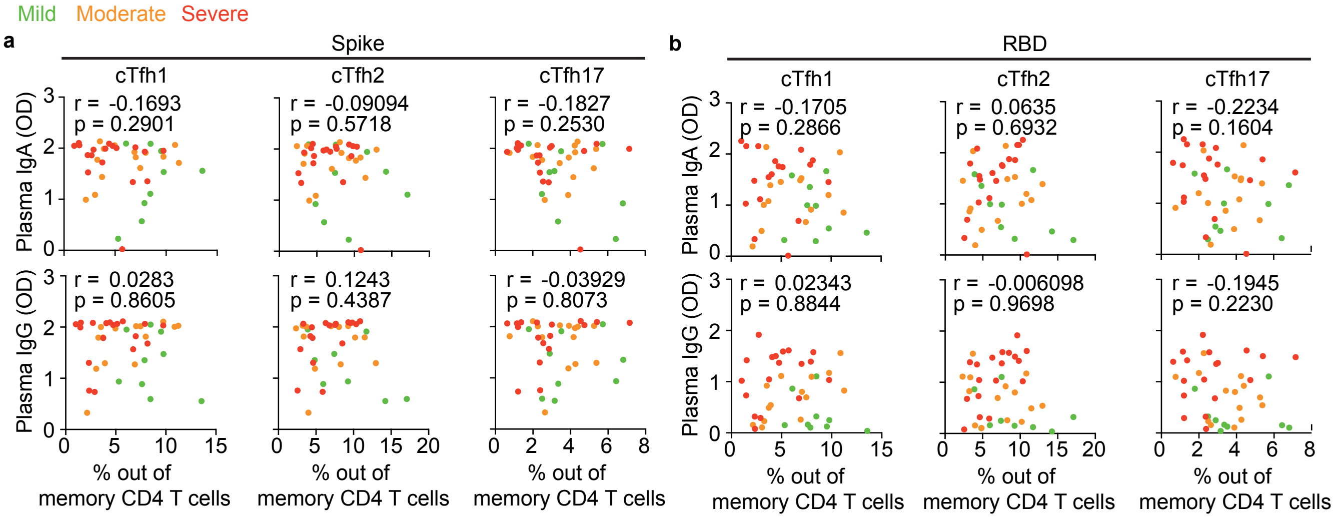

Supplementary Figure 2 Total frequencies of each cTfh cell subset did not correlate with serological IgA and IgG responses against SARS-CoV-2 Spike and RBD. Two-sided Spearman correlation for frequencies of each cTfh cell subset versus plasma immunoglobulins against the (a) spike and (b) RBD in COVID-19 patients (n=41) during acute infection. Dots are individual samples color-coded according to peak disease severity.

Supplementary figure 3

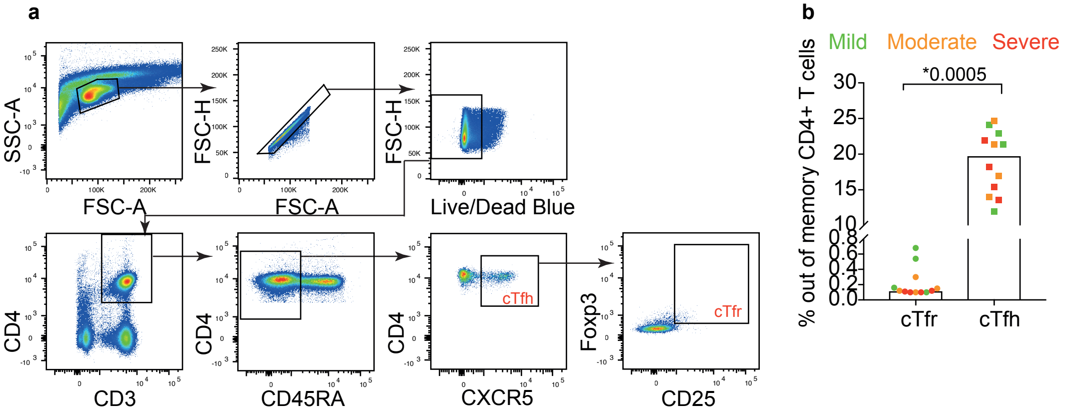

Supplementary Figure 3 Frequencies of circulating T follicular regulatory (cTfr) cells in COVID-19 patients (n=12) with acute SARS-CoV-2 infection. (a) Representative example with gating strategy to identify cTfr by flow cytometry. From single, live CD3^{+} CD4^{+} CD45RA^{-} CXCR5^{+} cTfh cells, CD25^{+} Foxp3^{+} cells were determined as cTfr. (b) Bar graphs show the individual frequencies of cTfr and cTfh cells out of memory CD4 T cells with median (n=12; n=4 mild, n=4 moderate and n=4 severe). Two-sided Wilcoxon matched-pairs signed test was used to consider statistically significant. P<0.05 was considered to be a significant difference *. P values <0.05 are listed above each comparison. Dots are individual samples color-coded according to peak disease severity.

Supplementary figure 4

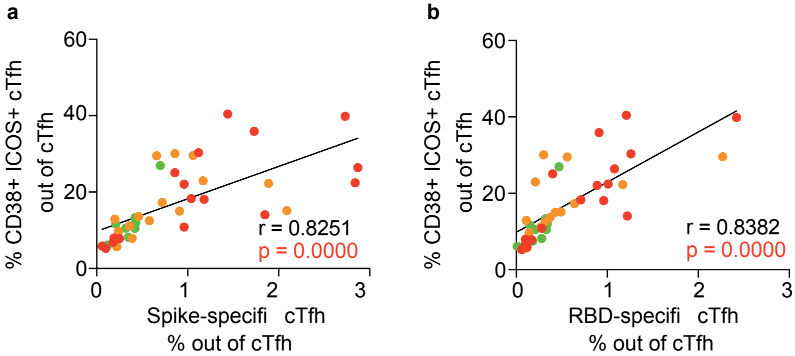

Supplementary Figure 4 Frequency of activated cTfh cells correlated with frequency of virus-specific cTfh cells during acute disease. Two-sided Spearman correlation for frequency of (a) spike-specific cTfh cells and (b) RBD-specific cTfh cells versus frequency of $CD38^{+}$ $ICOS^{+}$ cTfh cells. The dots are color-coded according to peak disease severity. 9, 14 and 18 individual samples from 9 mild, 14 moderate and 18 severe patients were analysed. For patients with longitudinal acute samples, data from the earliest sample was involved as representative in Spearman correlation analysis.

Supplementary figure 5

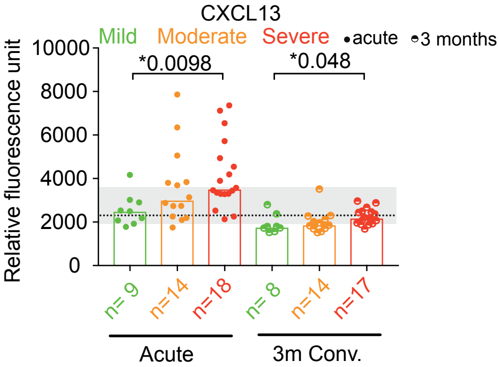

Supplementary Figure 5 Level of plasma CXCL13 in COVID-19 patients during acute disease and 3 months convalescence. Bar charts show the relative fluorescence unit (RFU) of plasma CXCL13 with median from COVID-19 patients with acute infection (Acute, full circle) and 3 months convalescence (3 months, half circle). Dotted line shows the median frequency with 95% CI (grey area) of healthy controls. For patients with longitudinal acute samples, data from the earliest sample was involved as representative. Two-sided Kruskal-Wallis with Dunn’s multiple comparisons test was used to assess statistical significance. P<0.05 was considered to be a significant difference *. P values are listed above each comparison.

Supplementary figure 6

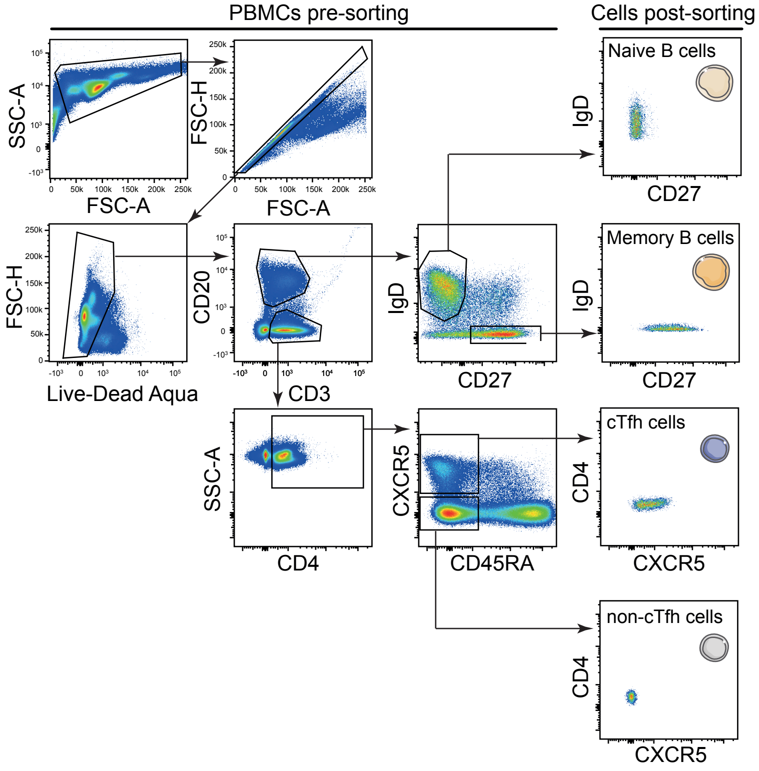

Supplementary Figure 6 Representative sorting strategy of cTfh, non-cTfh, memory and naïve B cells. Representative sorting strategy to isolate cTfh, non-cTfh, memory and naïve B cells from PBMC by flow cytometry. From single, live CD20$^-$ but CD3$^+$ CD4$^+$ T cells, memory CD4$^+$ T cells were identified as CD45RA$^-$. From memory CD4$^+$ T cells, CXCR5$^+$ cells were sorted and identified as cTfh cells and CXCR5$^-$ cells were sorted and identified as non-cTfh cells. From single, live CD3$^-$ but CD20$^+$ B cells, CD27$^-$ but IgD$^+$ and CD27$^+$ but IgD$^-$ cells were sorted and identified as naïve and memory B cells respectively.

Supplementary figure 7

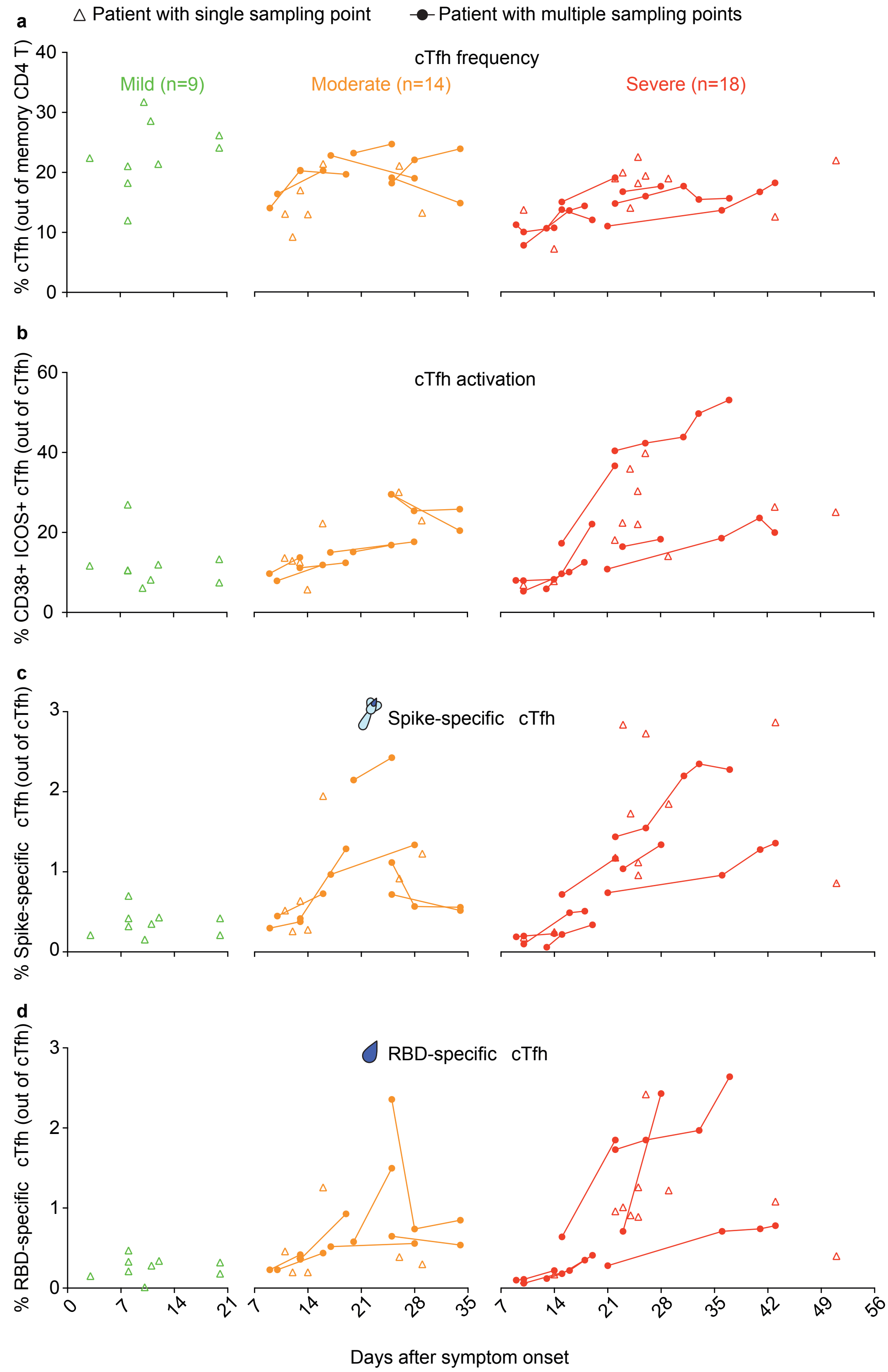

Supplementary Figure 7 Individual distribution of cTfh cells in individual COVID-19 patients across disease severity during acute infection. Figures show the longitudinal distribution of frequencies of (a) cTfh cells (b) activated cTfh cells (c) Spike-specific cTfh cells and (d) RBD-specific cTfh cells in individual COVID-19 patients across disease severity during acute infection. The dots are color-coded according to peak disease severity. Open triangles show patients with single sampling point, and dots linked with line show patients with multiple, longitudinal sampling points.

Supplementary figure 8

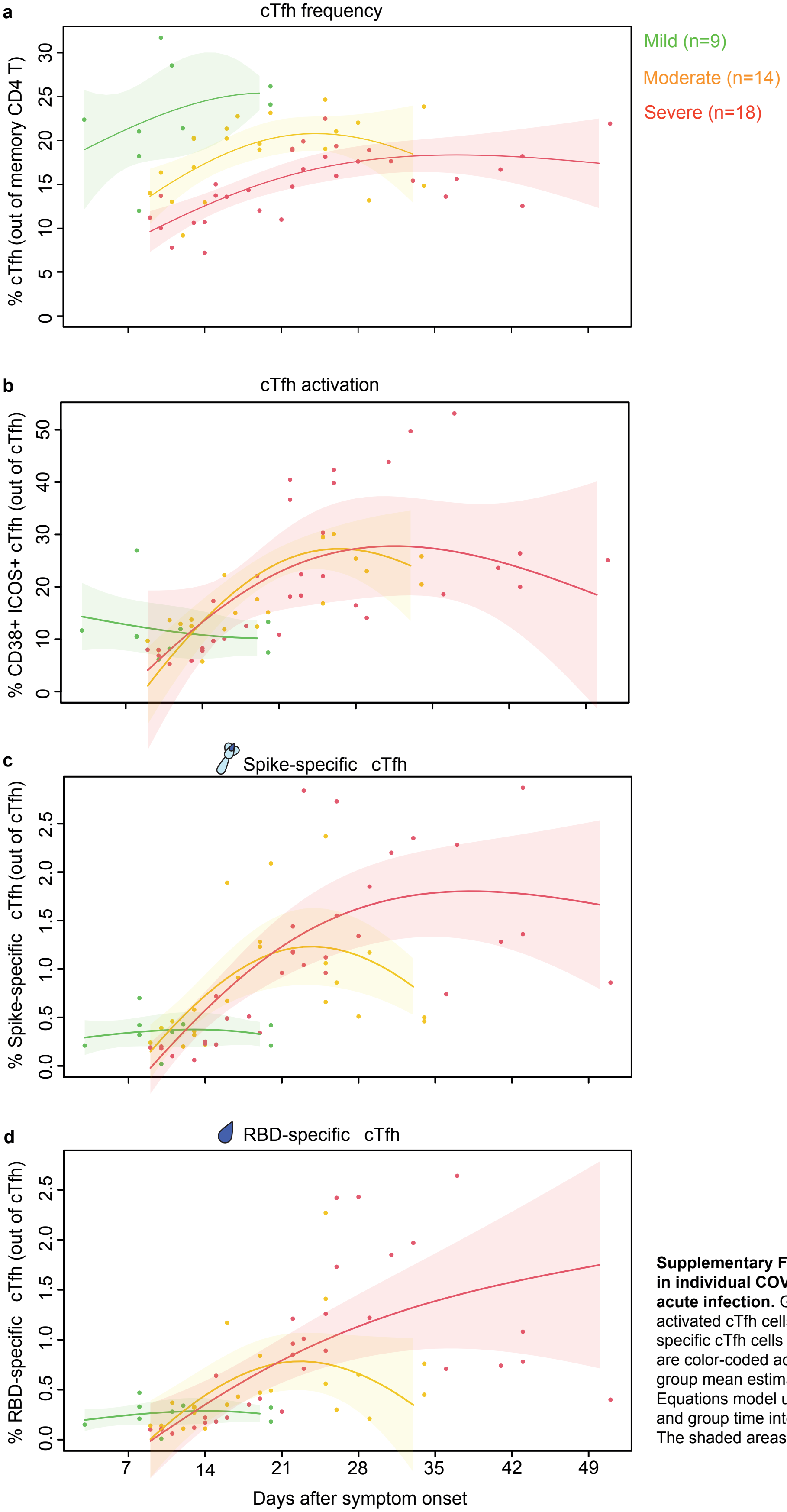

Supplementary Figure 8 Longitudinal distribution of cTfh cells in individual COVID-19 patients across disease severity during acute infection. Graphs show frequencies of (a) cTfh cells (b) activated cTfh cells (c) Spike-specific cTfh cells and (d) RBD-specific cTfh cells COVID-19 patients over time. The dots and lines are color-coded according to peak disease severity. Lines show group mean estimates based on two-sided Generalized Estimating Equations model using restricted cubic splines for the time effect and group time interaction for the estimation of group differences. The shaded areas are 95% CI.

Supplementary figure 9

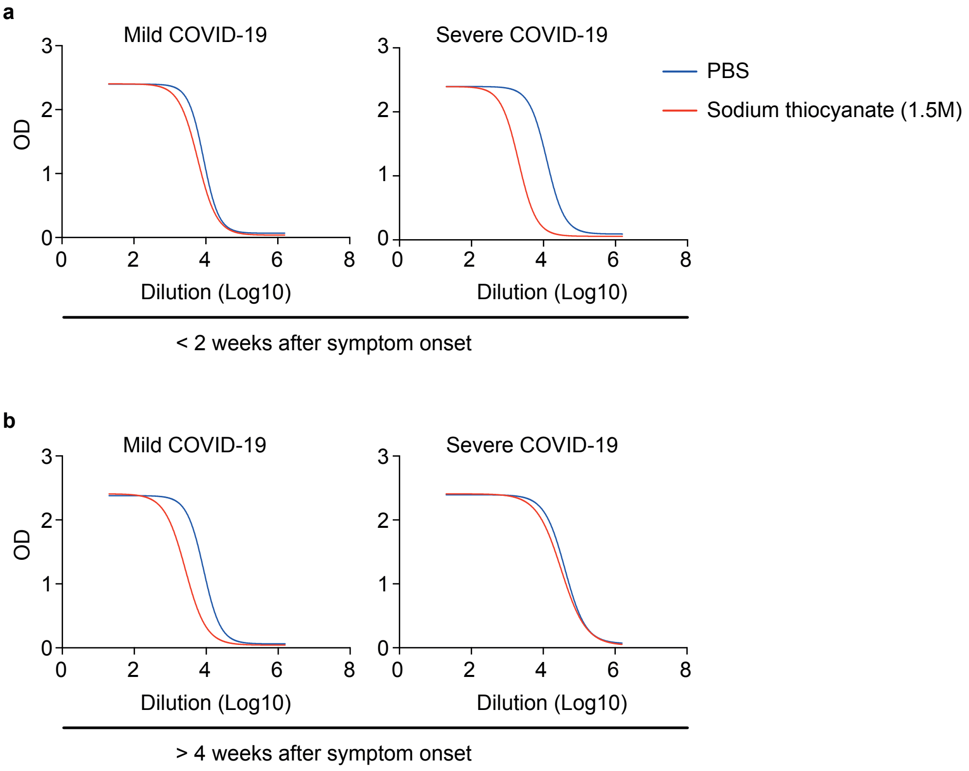

Supplementary Figure 9 Representative measurement of plasma IgG against SARS-CoV-2 spike avidity by ELISA. Representative plasma samples from mild and severe COVID-19 patients with symptom onset (a) less than 2 weeks and (b) over 4 weeks were tested by ELISA to determine the avidity of IgG against SARS-CoV-2 spike. Representative binding curves for plasma IgG against spike by ELISA in a 5-fold dilution series starting from 1:20 to 1:312500 and incubating with (Blue curve) PBS or (Red curve) 1.5M solution of sodium thiocyanate. EC50 have been calculated using non-linear fit of the data to a sigmoidal curve by constraining the top of each curve to the OD of 2.5.

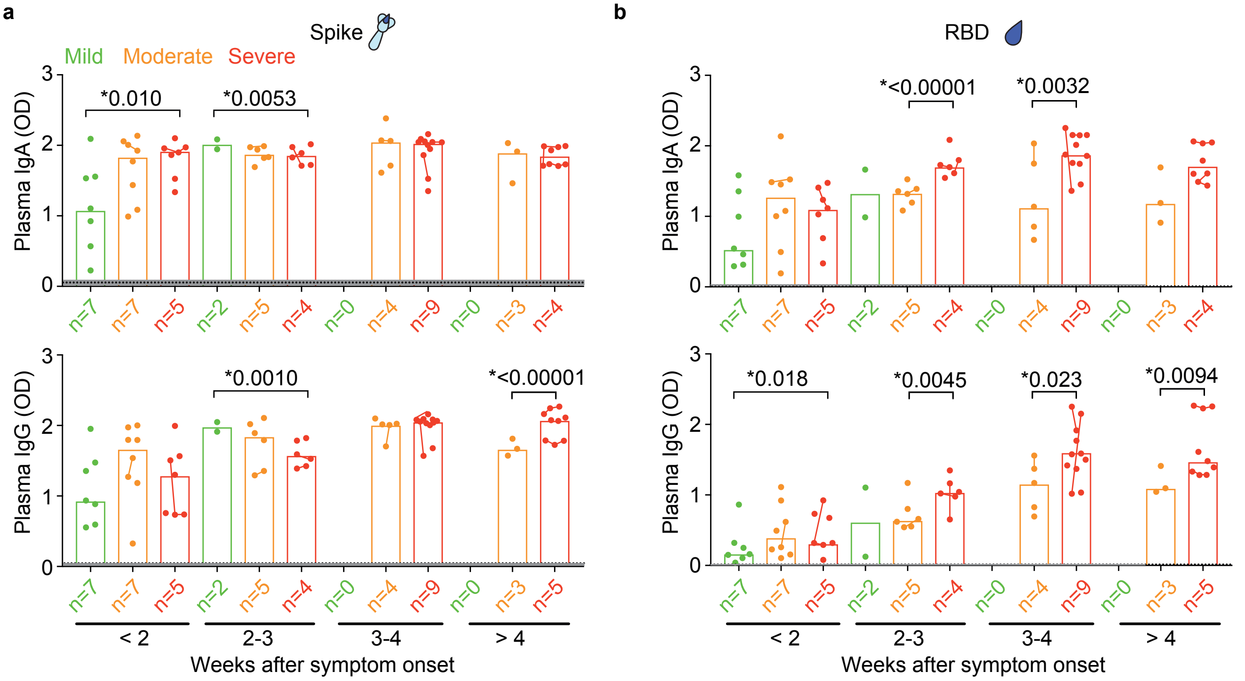

Supplementary Figure 10 Titers of plasma immunoglobulins against SARS-CoV-2 spike and RBD according to weeks after symptom onset. Titers of plasma IgA and IgG against SARS-CoV-2 (a) spike and (b) RBD were tested by ELISA and bar charts show the OD value with median in COVID-19 patients with acute disease according to weeks after symptom onset. Dots are individual samples color-coded according to peak disease severity. 9, 22 and 33 individual samples from 9 mild, 14 moderate and 18 severe patients respectively were analysed. X axis shows number of patients in each bar. One severe patient displaying IgA deficiency was excluded in all IgA analysis. Dots from same patient are linked with line in each bar. Dotted lines show the median frequency with 95% CI (grey area) of healthy controls. Time-period specific differences between groups were calculated using by using time-period specific subsets of the data. The graphical presentations of the different outcomes were based on a two-sided Generalized Estimating Equations model with time modelled using a restricted cubic spline with knots at 0, 14, 21, 28 and 53 days, without multiple comparisons. P<0.05 was considered to be a significant difference *. P values <0.05 are listed above each comparison.

Supplementary figure 11

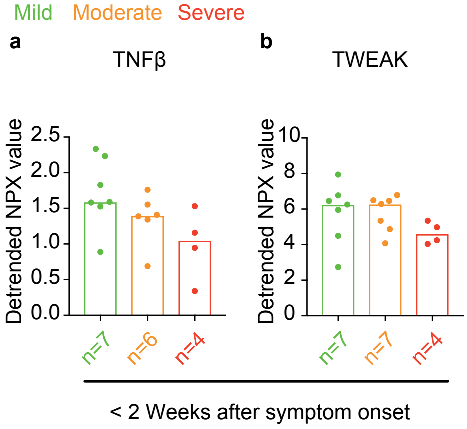

Supplementary Figure 11 Levels of plasma TNFβ and TWEAK in COVID-19 patients sampled during early SARS-CoV-2 infection (less than 2 weeks after symptom onset). Bar charts show the detrended NPX value of plasma (a) TNFβ and (b) TWEAK with median from COVID-19 patients during less than 2 weeks after symptom onset. For patients with longitudinal acute samples, data from the earliest sample was involved as representative. X axis shows number of patients in each bar. Two-sided Kruskal-Wallis with Dunn's multiple comparisons test was used to assess statistical significance. $P<0.05$ was considered to be a significant difference *.

Supplementary figure 12

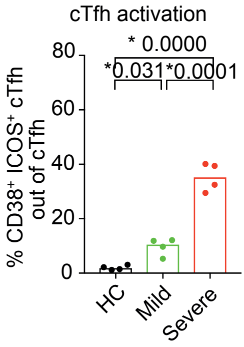

Supplementary Figure 12 Frequency of activated cTfh cells in COVID-19 patients and healthy donors selected in cTfh functional assay. Bar charts show the frequency of activated cTfh cells with mean in COVID-19 patients with acute disease and healthy donors selected in cTfh functional assay. One-Way ANOVA was used to consider statistically significant. $P<0.05$ was considered to be a significant difference *. P values <0.05 are listed above each comparison.

Supplementary Table 1. Antibodies for cTfh cells phenotyping.

Phenotyping of cTfh cells					
Fluorochrome	Marker	Company	Clone	Dilution	Catalog number
PerCP-Cy5.5	CD4	Biolegend	OKT4	3:100	317428
FITC	CD62L	Biolegend	DREF-56	3:100	304804
PE-Cy7	ICOS	Biolegend	C398-4A	1:100	313520
PE-Cy5	CD40L	Biolegend	24-31	3:100	310808
PE-TR	CXCR5	Invitrogen	MU5UBEE	3:100	61-9185-42
PE	CD38	Biolegend	HIT2	1:200	303505
APC-Cy7	CD3	BD Biosciences	SK7	3:100	345767
AF700	CCR7	Biolegend	G043H7	3:100	353244
BV786	CCR6	BD Biosciences	11A9	3:100	563704
BV650	CXCR3	Biolegend	G025H7	1:25	353730
BV421	PD1	BD Biosciences	EH12.1	1:50	565935
BUV395	CD45RA	BD Biosciences	HI100	1:100	740298

Supplementary Table 2. Antibodies for B cells phenotyping.

Phenotyping of B cells					
Fluorochrome	Marker	Company	Clone	Dilution	Catalog number
PerCP-Cy5.5	CD38	BD Biosciences	HIT2	3:100	551400
PE-Cy7	CD19	BD Biosciences	HIB19	3:100	560728
APC-Cy7	CD20	BD Biosciences	2H7	1:25	560734
BV786	IgG	BD Biosciences	G18-145	1:20	564230
BV650	CD27	BD Biosciences	M-T271	3:100	564894
BV510	CD14	Biolegend	M5E2	1:50	301842
BV510	CD16	BD Biosciences	3G8	1:50	563830
BV510	CD56	BD Biosciences	NCAM-1	3:200	740171
BUV 395	IgM	BD Biosciences	G20-217	3:200	563903
BUV 395	IgD	BD Biosciences	IA6-2	1:50	563813

Supplementary Table 3. Antibodies for SARS-CoV-2-specific cTfh cells phenotyping.

Phenotyping of SARS-CoV-2-specific cTfh cells					
Fluorochrome	Marker	Company	Clone	Dilution	Catalog number
PerCP-Cy5.5	CD134	BD Biosciences	ACT35	1:40	563659
PE-TR	CXCR5	Invitrogen	MU5UBEE	3:100	61-9185-42
APC-Cy7	CD3	BD Biosciences	SK7	3:100	345767
AF700	CD4	BD Biosciences	L200	1:50	560836
APC	CD25	Biolegend	BC96	3:100	302610
BV786	CCR6	BD Biosciences	11A9	3:100	563704
BV650	CXCR3	Biolegend	G025H7	1:25	353730
BUV395	CD45RA	BD Biosciences	HI100	1:100	740298

Supplementary Table 4. Antibodies for cTfh and B cells sorting.

cTfh and B cells sorting					
Fluorochrome	Marker	Company	Clone	Dilution	Catalog number
FITC	IgD	BD Biosciences	IA6-2	1:50	562023
PE	CD3	BD Biosciences	SK7	1:20	345765
PE-Cy5	CD45RA	BD Biosciences	5H9	1:100	552888
PE-TR	CXCR5	Invitrogen	MU5UBEE	3:100	61-9185-42
APC-Cy7	CD20	BD Biosciences	2H7	1:25	560734
AF700	CD4	BD Biosciences	L200	1:50	560836
BV650	CD27	BD Biosciences	M-T271	3:100	564894

Supplementary Table 5. Demographic and clinical information of COVID-19 patients involved in in vitro co-culture assay.

Peak Disease severity	Mild	Severe	Significance[A]
Number of Individuals	4	4	
Age in years, mean ± SD (range)	55 ± 3.7 (51-60)	68 ± 5.4 (24-81)	P = 0.0286
Male, n (%)	2 (50)	4 (100)	P = 0.4286
Female, n (%)	2 (50)	0 (0)	
Days after onset of symptom, mean ± SD (range)	11 ± 1.3 (9-12)	34 ± 14.6 (18-53)	P = 0.0286

[A] Two-sided Mann-Whitney U unpaired t-test was performed to determine statistical significance.
