# Supplementary material for: Delayed generation of functional virus-specific circulating T follicular helper cells correlates with severe COVID-19
Source: Nat Commun. 2023 Apr 15;14:2164. doi: 10.1038/s41467-023-37835-9 (PMC10105364; doi:10.1038/s41467-023-37835-9)
Supplement: Supplementary file 3 — Reporting Summary [file 41467_2023_37835_MOESM3_ESM.pdf]

nature portfolio

Corresponding author(s): Anna Smed-Sörensen
Last updated by author(s): Mar 18, 2023

Reporting Summary

Nature Portfolio wishes to improve the reproducibility of the work that we publish. This form provides structure for consistency and transparency in reporting. For further information on Nature Portfolio policies, see our Editorial Policies and the Editorial Policy Checklist.

Statistics

For all statistical analyses, confirm that the following items are present in the figure legend, table legend, main text, or Methods section.

n/a	Confirmed	
☐	☒	The exact sample size (*n*) for each experimental group/condition, given as a discrete number and unit of measurement
☐	☒	A statement on whether measurements were taken from distinct samples or whether the same sample was measured repeatedly
☐	☒	The statistical test(s) used AND whether they are one- or two-sided<br>*Only common tests should be described solely by name; describe more complex techniques in the Methods section.*
☒	☐	A description of all covariates tested
☐	☒	A description of any assumptions or corrections, such as tests of normality and adjustment for multiple comparisons
☐	☒	A full description of the statistical parameters including central tendency (e.g. means) or other basic estimates (e.g. regression coefficient) AND variation (e.g. standard deviation) or associated estimates of uncertainty (e.g. confidence intervals)
☐	☒	For null hypothesis testing, the test statistic (e.g. *F*, *t*, *r*) with confidence intervals, effect sizes, degrees of freedom and *P* value noted<br>*Give P values as exact values whenever suitable.*
☒	☐	For Bayesian analysis, information on the choice of priors and Markov chain Monte Carlo settings
☒	☐	For hierarchical and complex designs, identification of the appropriate level for tests and full reporting of outcomes
☒	☐	Estimates of effect sizes (e.g. Cohen's *d*, Pearson's *r*), indicating how they were calculated

Our web collection on statistics for biologists contains articles on many of the points above.

Software and code

Policy information about availability of computer code

Data collection	Bio-Plex 200 analyzer (BIO-RAD) was used to collect cytokine data. LSR Fortessa flow cytometer (BD) was used to collect Flow cytometry data. FACS Aria Fusion (BD) was used to sort cTfh, non-cTfh and B cells.
Data analysis	FlowJo 10 was used to analyze Flow cytometry data. RStudio (version 4.1.2), Prism 9 (GraphPad) and SPSS version 27.0 (IBM, New York) were used to perform statistical analysis.

For manuscripts utilizing custom algorithms or software that are central to the research but not yet described in published literature, software must be made available to editors and reviewers. We strongly encourage code deposition in a community repository (e.g. GitHub). See the Nature Portfolio guidelines for submitting code & software for further information.

Data

Policy information about availability of data

All manuscripts must include a data availability statement. This statement should provide the following information, where applicable:
- Accession codes, unique identifiers, or web links for publicly available datasets
- A description of any restrictions on data availability
- For clinical datasets or third party data, please ensure that the statement adheres to our policy

The personal data are not publicly available due to them containing information that could compromise research participant privacy. All other data are provided in the article and its Supplementary files or from the corresponding author upon request. Source data are provided with this paper.

Human research participants

Policy information about studies involving human research participants and Sex and Gender in Research.

Reporting on sex and gender	COVID-19 patients confirmed by SARS-CoV-2 PCR test were recruited for this study, including both male and female patients. 20 age and gender matched healthy donors recruited prior to the pandemic were also included as healthy controls for this study.
Population characteristics	Among the 49 COVID-19 patients, all were adults with an age range of 26-77 years (median age of 55 years), with females comprising 33%. All COVID-19 patients were confirmed by positive RT-PCR results for SARS-CoV-2. The 20 healthy donors were selected aiming to have similar age and gender as the COVID-19 patient cohort. Among the 20 healthy donors, all were adults with an age range of 24-81 years (median age of 53 years), with 30% of them being females.
Recruitment	COVID-19 patients confirmed by SARS-CoV-2 PCR test were enrolled in March-May 2020 at the Karolinska University Hospital and Haga Outpatient Clinic (Haga Närakut), Stockholm, Sweden. Additionally, patients with mild disease were included by recruiting household contacts who were SARS-CoV-2 PCR positive. In total 147 COVID-19 adult patients were recruited.<br><br>For this study, we selected patients with non-fatal outcome and available biobanked longitudinal acute and convalescent samples, and excluded patients with autoimmune disease or haematological malignancies. It has been shown that cTfh cells display aberrant phenotype or functionality in patients with autoimmune disease or haematological malignancies. We excluded these patients to make sure that the characteristics we observed in cTfh cells were due to SARS-CoV-2 infection, not other disease. We included all available patients who fit to the requirements described above, and consequently, 41 COVID-19 patients were included in this study. All 20 healthy donors were recruited in Sweden before November 2019, so none of them had been exposed to SARS-CoV-2. These healthy donors were selected based on their age and gender to be similar to the COVID-19 patient cohort, as a control group.<br><br>The data in figures 1,2,3 and 5, supplementary figures 1,2,3,4,5,7,8,9 and 10 were generated from the 41 COVID-19 patients and 20 healthy donors described above. Thus how these patient and healthy donor cohort were selected would not introduce any biases in this study.<br><br>The functional co-culture experiments in figure 4, supplementary figures 6 and 11, required large amount of cells. Our samples biobanked in the first wave of the COVID-19 pandmic (March-May 2020) were not sufficient. So we recruited additional 8 COVID-19 patients (4 mild and 4 severe) in December 2020 – March 2021, who were confirmed with SARS-CoV-2 infection by PCR, and were able to donate 40 mL blood, and did not have autoimmune disease or haematological malignancies. The recruitment of these patients did not introduce any bias in this study.
Ethics oversight	The study was approved by the Swedish Ethical Review Authority, and performed according to the Declaration of Helsinki. Written informed consent was obtained from all patients and controls. For sedated patients, the denoted primary contact was contacted and asked about the presumed will of the patient and to give initial oral and subsequently signed written consent. When applicable, retrospective written consent was obtained from patients with non-fatal outcomes.

Note that full information on the approval of the study protocol must also be provided in the manuscript.

Field-specific reporting

Please select the one below that is the best fit for your research. If you are not sure, read the appropriate sections before making your selection.

☒ Life sciences ☐ Behavioural & social sciences ☐ Ecological, evolutionary & environmental sciences

For a reference copy of the document with all sections, see nature.com/documents/nr-reporting-summary-flat.pdf

Life sciences study design

All studies must disclose on these points even when the disclosure is negative.

Sample size	With a sample size of n = 20 per group, the probability is 80% that the study will detect a relationship between the independent and the dependent variables at a two-sided 0.05 significance level, if the true change in the dependent variables is 0.663 standard deviations per one standard deviation change in the independent variable.<br><br>In this study, we included as many patients who fit the requirements described in the Recruitment, and consequently 49 COVID-19 patients were included in the patient cohort, which is approximately twice as high as the power analysis. 20 age and gender matched healthy donors were selected as a control cohort, which fits the requirement of power analysis.
Data exclusions	All samples from all study participants were included in the analysis of this study, except one severe patient displayed IgA deficiency over time was excluded in all IgA analysis.
Replication	Independently replication was performed in antibody ELISA, virus neutralization assay, cytokines/chemokines proteomics assay with plasma sample from patient and healthy control cohort. Given limited cell sample availability, replication was not performed in flow cytometry or

	Luminex assay.
Randomization	In this study, all patients were grouped based on their peak COVID-19 disease severity according to the respiratory domain of the sequential organ failure assessment (SOFA) score.
Blinding	Investigators were not blinded. In each single experiment, samples from COVID-19 patient cohort and healthy control cohort were run in parallel, to make sure the difference observed between patient and control cohort were not induced by different batches of experiments. The data generation for samples collected from acute disease and convalescence from the same patient were also run in parallel in single experiments, to make sure the difference observed between acute disease and convalescence were not induced by different batches of experiments.

Reporting for specific materials, systems and methods

We require information from authors about some types of materials, experimental systems and methods used in many studies. Here, indicate whether each material, system or method listed is relevant to your study. If you are not sure if a list item applies to your research, read the appropriate section before selecting a response.

Materials & experimental systems

n/a	Involved in the study	
☐	☒	Antibodies
☒	☐	Eukaryotic cell lines
☒	☐	Palaeontology and archaeology
☒	☐	Animals and other organisms
☒	☐	Clinical data
☒	☐	Dual use research of concern

Methods

n/a	Involved in the study	
☒	☐	ChIP-seq
☐	☒	Flow cytometry
☒	☐	MRI-based neuroimaging

Antibodies

Antibodies used

Phenotyping of cTfh cells:
PerCP-Cy5.5 CD4 Biolegend, OKT4 Cat#317428 Dilution3:100
FITC CD62L Biolegend, DREF-56 Cat#304804 Dilution3:100
PE-Cy7 ICOS Biolegend, C398-4A Cat#313520 Dilution1:100
PE-Cy5 CD40L Biolegend, 24-31 Cat#310808 Dilution3:100
PE-TR CXCR5 Invitrogen, MU5UBEE Cat#61-9185-42 Dilution3:100
PE CD38 Biolegend, HIT2 Cat#303505 Dilution1:200
APC-Cy7 CD3 BD Biosciences, SK7 Cat#345767 Dilution3:100
AF700 CCR7, Biolegend, G043H7 Cat#353244 Dilution3:100
BV786 CCR6, BD Biosciences, 11A9 Cat#563704 Dilution3:100
BV650 CXCR3, Biolegend, G025H7 Cat#353730 Dilution1:25
BV421 PD1, BD Biosciences, EH12.1 Cat#565935 Dilution1:50
BUV395 CD45RA, BD Biosciences, HI100 Cat#740298 Dilution1:100
Phenotyping of B cells:
PerCP-Cy5.5 CD38, BD Biosciences, HIT2 Cat#551400 Dilution3:100
PE-Cy7 CD19, BD Biosciences, HIB19 Cat#560728 Dilution3:100
APC-Cy7 CD20, BD Biosciences, 2H7 Cat#560734 Dilution1:25
BV786 IgG, BD Biosciences, G18-145 Cat#564230 Dilution1:20
BV650 CD27, BD Biosciences, M-T271 Cat#564894 Dilution3:100
BV510 CD14, Biolegend, M5E2 Cat#301842 Dilution1:50
BV510 CD16, BD Biosciences, 3G8 Cat#563830 Dilution1:50
BV510 CD56, BD Biosciences, B159 Cat#740171 Dilution3:200
BUV 395 IgM, BD Biosciences, G20-217 Cat#563903 Dilution3:200
BUV 395 IgD, BD Biosciences, IA6-2 Cat#563813 Dilution1:50
Phenotyping of SARS-CoV-2-specific cTfh cells:
PerCP-Cy5.5 CD134, BD Biosciences, ACT35 Cat#563659 Dilution1:40
PE-TR CXCR5, Invitrogen, MU5UBEE Cat#61-9185-42 Dilution3:100
APC-Cy7 CD3, BD Biosciences, SK7 Cat#345767 Dilution3:100
AF700 CD4, BD Biosciences, L200 Cat#560836 Dilution1:50
APC CD25, Biolegend, BC96 Cat#302610 Dilution3:100
BV786 CCR6, BD Biosciences, 11A9 Cat#563704 Dilution3:100
BV650 CXCR3, Biolegend, G025H7 Cat#353730 Dilution1:25
BUV395 CD45RA, BD Biosciences, HI100 Cat#740298 Dilution1:100
cTfh and B cells sorting:
FITC IgD, BD Biosciences, IA6-2 Cat#562023 Dilution1:50
PE CD3, BD Biosciences, SK7 Cat#345765 Dilution1:20
PE-Cy5 CD45RA, BD Biosciences, 5H9 Cat#552888 Dilution1:100

	PE-TR CXCR5, Invitrogen, MU5UBEE Cat#61-9185-42 Dilution3:100<br>APC-Cy7 CD20, BD Biosciences, 2H7 Cat#560734 Dilution1:25<br>AF700 CD4, BD Biosciences, L200 Cat#560836 Dilution1:50<br>BV650 CD27, BD Biosciences, M-T271 Cat#564894 Dilution3:100
Validation	All antibodies are validated by their respective manufacturers for the application of flow cytometric analysis. All antibodies have been titrated in the lab and validated to bind to the correct antigen by staining negative and positive control cells by flow cytometric analysis before use. For more information on the antibodies used, please visit biolegend.com, bdbiosciences.com and themofisher.com.

Flow Cytometry

Plots

Confirm that:

- [x] The axis labels state the marker and fluorochrome used (e.g. CD4-FITC).
- [x] The axis scales are clearly visible. Include numbers along axes only for bottom left plot of group (a 'group' is an analysis of identical markers).
- [x] All plots are contour plots with outliers or pseudocolor plots.
- [x] A numerical value for number of cells or percentage (with statistics) is provided.

Methodology

Sample preparation	Biobanked PBMCs and plasma were isolated from blood of all study participants.
Instrument	Bio-Plex 200 analyzer (BIO-RAD) was used to collect cytokines data. LSR Fortessa flow cytometer (BD) was used to collect Flow cytometry data. FACS Aria Fusion (BD) was used to isolate cTfh, non-cTfh and B cells.
Software	FlowJo 10 was used to analyze Flow cytometry data. RStudio (version 4.1.2), Prism 9 (GraphPad) and SPSS version 27.0 (IBM, New York) were used to perform statistical analysis.
Cell population abundance	50 to 70 millions of PBMCs from 8 patients with SARS-CoV-2 infection were prepared to sort cTfh cells, non-cTfh cells, memory B cells and naive B cells using flow cytometry. The purities of isolated cell populations are over 99%.
Gating strategy	From single, live CD20- but CD3+ CD4+ T cells, memory CD4+ T cells were identified as CD45RA-. From memory CD4+ T cells, CXCR5+ cells were identified as cTfh cells and CXCR5- cells were identified as non-cTfh cells. From single, live CD3- but CD20+ B cells, CD27- but IgD+ and CD27+ but IgD- cells were identified as naïve and memory B cells respectively.

- [x] Tick this box to confirm that a figure exemplifying the gating strategy is provided in the Supplementary Information.
